# Natural history of large regenerative nodules and dysplastic nodules in liver cirrhosis: 28-year follow-up study

**DOI:** 10.1007/s12072-015-9620-6

**Published:** 2015-03-03

**Authors:** Tsunenobu Sato, Fukuo Kondo, Masaaki Ebara, Nobuyuki Sugiura, Shinichiro Okabe, Masahiko Sunaga, Masaharu Yoshikawa, Eiichiro Suzuki, Sadayuki Ogasawara, Yusuke Shinozaki, Yoshihiko Ooka, Tetsuhiro Chiba, Fumihiko Kanai, Takashi Kishimoto, Yukio Nakatani, Toshio Fukusato, Osamu Yokosuka

**Affiliations:** 1 Department of Gastroenterology and Nephrology, Graduate School of Medicine, Chiba University, 1-8-1 Inohana, Chuo-ku, Chiba City, Chiba 260-8670 Japan; 2 Department of Gastroenterology, Chiba Central Medical Center, 1835-1 Kasoricho, Wakaba-ku, Chiba City, Chiba 264-0017 Japan; 3 Department of Pathology, Teikyo University Hospital, 2-11-1 Kaga, Itabashi-ku, Tokyo, 173-8606 Japan; 4 Department of Gastroenterology, Matsudo City Hospital, 4005 Kamihongo, Matsudo City, Chiba 271-8511 Japan; 5 Department of Internal Medicine, National Hospital Organization Chiba Medical Center, 4-1-2 Tsubakimori, Chuo-ku, Chiba City, Chiba 260-8606 Japan; 6 Department of Molecular Pathology, Graduate School of Medicine, Chiba University, 1-8-1 Inohana, Chuo-ku, Chiba City, Chiba 260-8670 Japan; 7 Department of Diagnostic Pathology, Graduate School of Medicine, Chiba University, 1-8-1 Inohana, Chuo-ku, Chiba City, Chiba 260-8670 Japan; 8 Department of Pathology, School of Medicine, Teikyo University, 2-11-1 Kaga, Itabashi-ku, Tokyo, 173-8605 Japan

**Keywords:** Large regenerative nodule, Dysplastic nodule, Natural history, Malignant transformation

## Abstract

**Background and aims:**

Some follow-up studies of large regenerative nodules (LRNs) and dysplastic nodules (DNs) were reported previously. However, the pre-malignant potentiality of LRNs has remained controversial up to now. No LRNs showed malignant transformation in our previous study. We aimed to evaluate the pre-malignant potentiality of LRNs and DNs with a greater number of cases and longer follow-up periods.

**Methods:**

From 1982 to 2005, 1,500 consecutive nodular lesions up to 2 cm in diameter were subjected to US guided thin-needle biopsy in cirrhotic patients at Chiba University Hospital. Of these lesions, 68 LRNs in 60 cases and 20 DNs in 22 cases were followed up for more than 6 months without any anti-cancer therapy. The last US examination was in 2010. The total study period was 28 years. We analyzed the histological findings and the clinical data of all cases retrospectively. The outcome of the lesions was examined.

**Results:**

The mean follow-up period was 38.9 (16–119) months in LRNs and 31.9 (6–101 months) in DNs. Rate of nodule enlargement was higher in DNs (8/24 nodules, 33 %) than LRNs (11/68 nodules, 16 %), (*p* = 0.0743, not significant). Rate of malignant transformation was also higher in DNs (10/24 nodules, 42 %) than LRNs (9/68 nodules, 13 %), (*p* = 0.0040, significant). The rate of disappearance in images was similar between LRNs and DNs.

**Conclusions:**

We should recognize LRN as low risk pre-malignant lesions whereas DNs as high risk lesions.

## Introduction

Although several follow-up studies of large regenerative nodules (LRNs) and dysplastic nodules (DNs) have been reported [1–9], the pre-malignant potentiality of LRNs is still controversial. No LRNs showed malignant transformation in our previous study [1], whereas some authors reported that LRNs were pre-malignant [3, 8, 9]. However, these earlier reports are not easily compared, as the histological criteria of well differentiated hepatocellular carcinoma (HCC), DN and LRN were lacking universal consensus at that time [10, 11]. In addition, the numbers of nodules were not so many and the follow-up periods were not so long. A few reports studied over 90 nodules [8, 9], with the others studied less than 40 nodules [1–6]. In four of the nine previous studies, the longest follow-up period was less than 5 years. If the numbers of nodules and the lengths of the follow-up periods had been greater, the results might also have been different.

In the present study, we studied many more LRNs than in our previous study, and the follow-up period was also elongated. DNs were also studied and compared with the results of LRNs. This is the only study which dealt with more than 90 nodules smaller than 2 cm in which the longest follow-up period was more than 5 years.

## Materials and methods

From 1982 to 2005, 1500 consecutive nodular lesions up to 2 cm in diameter were subjected to ultrasound (US) guided thin-needle biopsy in cirrhotic patients at Chiba University Hospital. They were diagnosed as 898 HCCs, 108 DNs, 315 LRNs, and 179 other lesions. Of the 108 DNs, 76 DNs were treated according to the patient’s preference or were confirmed as HCC by imaging diagnosis within 6 months. The remaining 32 DNs were followed up without any anti-cancer therapy. Informed consent of liver biopsy was taken from each patient. Within 6 months, eight DNs dropped out. Finally, 24 DNs from 22 cases were followed up for longer than 6 months. Similarly, 68 LRNs from 60 cases were followed up for more than 6 months. The last US examination of this study was performed in 2010, meaning that the total study period was 28 years (1982–2010). At this final point, we analyzed the histological findings and the clinical data of all cases retrospectively.

The outcome of the lesions (i.e., no change, enlargement of nodule size, malignant transformation and disappearance in images) was examined.

Profiles of the cases and nodules are shown in Tables 1 and 2, including age, male/female ratio, pre-existing HCC nodules, single or multiple lesions, diabetes mellitus (DM), liver function tests and tumor markers. No significant differences were seen between LRNs and DNs in respect to these items. As to the causes of cirrhosis in LRNs and DNs, hepatitis C virus was the most common, followed by hepatitis B virus. Cases of alcoholic cirrhosis were not included in this study (Table 1). Median nodule size was 12 mm in both LRNs and DNs (Table 2). Findings of ultrasound, dynamic CT and MRI were not statistically different between LRNs and DNs.

**Table 1:** Profile of the cases

Characteristic	Large regenerative nodules (LRNs)	Dysplastic nodules (DNs)	*p* value
Nodule, *n*	68	24	
Case, *n*	60	22	
Age	66 (24–79)	63 (37–71)	0.6080^a^
Male/female	39/21	18/4	0.1815^b^
Pre-HCC (+)/(−)	10/44	1/21	0.2730^b^
Single nodule case, *n*	55	21	1.0000^b^
Multiple nodule case, *n*	5	1	
Etiology			0.8677^c^
HCV	44	16	
HBV	8	4	
HCV and HBV	1	0	
Alcohol	0	0	
Others	7	2	
DM (+)/(−)	7/53	3/19	1.0000^b^
Child classification A/B/C	53/4/3	19/1/2	0.7529^c^
AST (IU/ml)	72 (15–400)	57 (34–197)	0.6787^a^
ALT (IU/ml)	69 (11–466)	63 (27–307)	0.5641^a^
Platelet (×10^4^/μl)	11.0 (3.7–20.9)	12.1 (5.5–21.0)	0.2231^a^
AFP (ng/ml)	8.2 (1.8–657)	10.5 (3.7–55.3)	0.5040^a^
PIVKA-II (mAU/ml)	18 (< 10–31)	15 (< 10–24)	0.1755^a^

Data are shown as median and range

^a^Mann–Whitney test

^b^Fisher’s exact test

^c^Chi-square test

**Table 2:** Profile of nodule

Parameter	Large regenerative nodules (LRNs)	Dysplastic nodules (DNs)	*p* value
Nodule, *n*	68	24	
Median size (diameter, mm)	12 (8–20)	12 (8–17)	0.8891^a^
US, hypo/hyper	53/15	14/10	0.1071^b^
Dynamic CT			
Plain phase, high/iso/low	1/58/6	0/18/4	0.4521^c^
Arterial phase, high/iso/low	4/54/7	0/19/5	0.2434^c^
Late phase, high/iso/low	2/52/11	0/19/5	0.6411^c^
MRI			
T1WI, high/iso/low	7/33/2	4/7/0	0.2996^c^
T2WI, high/iso/low	6/33/3	0/10/1	0.4109^c^

^a^Mann–Whitney test

^b^Fisher’s exact test

^c^Chi-square test

### Measurement of nodule size

The longer diameter of the nodule shown on the monitor of the ultrasound (US) was measured. We used a pre-installed measuring tool in the US instrument. To avoid a measurement error, more than one doctor performed US examination. As a principle, one of them was a well-experienced (more than 10 years) doctor. In case the longer diameter increased 1.5 times the former size, we interpreted that the diameter increased significantly.

### Definition of malignant transformation

Histological and/or imaging features which suggest HCC were used as the criteria of malignant transformation.

We evaluated nodule enlargement and malignant transformation independently. A nodule which showed enlargement of the size without malignant features in histological and imaging studies was simply interpreted as enlargement, whereas a nodule which showed malignant changes without enlargement was classified as malignant transformation.

### Statistical analysis

In order to compare the various data of LRNs and DNs, the following statistical methods were used.

- *Mann–Whitney test*: median age, median nodule size, AST, ALT, platelet, AFP, PIVKA-II
- *Fisher’s exact test*: male/female ratio, pre-existing HCC nodules, single or multiple lesions, US findings of nodules, diabetes mellitus
- *Chi*-*square test*: etiology of cirrhosis, CT and MRI findings of nodules, occurrence of new HCC lesions remote from nodules, Child classification
- *Log*-*rank (Mantel*–*Cox) test*: rate of nodule enlargement, rate of progression to malignancy, rate of disappearance on ultrasonography
- *p* value less than 0.05 was recognized as significant.

## Results

The mean follow-up period was 38.9 (16–119) months for LRNs and 31.9 (6–101 months) for DNs. Of the 68 LRNs, 11 showed enlargement and 35 became undetectable on US. Of the 24 DNs, eight showed enlargement and eight disappeared on US. All of the LRNs and DNs which disappeared remained undetectable by other types of imaging (CT and MRI) during the additional follow-up periods. As for the disappeared nodules, HCCs were not found in their loci. Finally, malignant transformation was proven in nine LRNs and ten DNs.

### Comparison of outcomes of LRNs and DNs

#### Rate of nodule enlargement

**Fig. 1:**
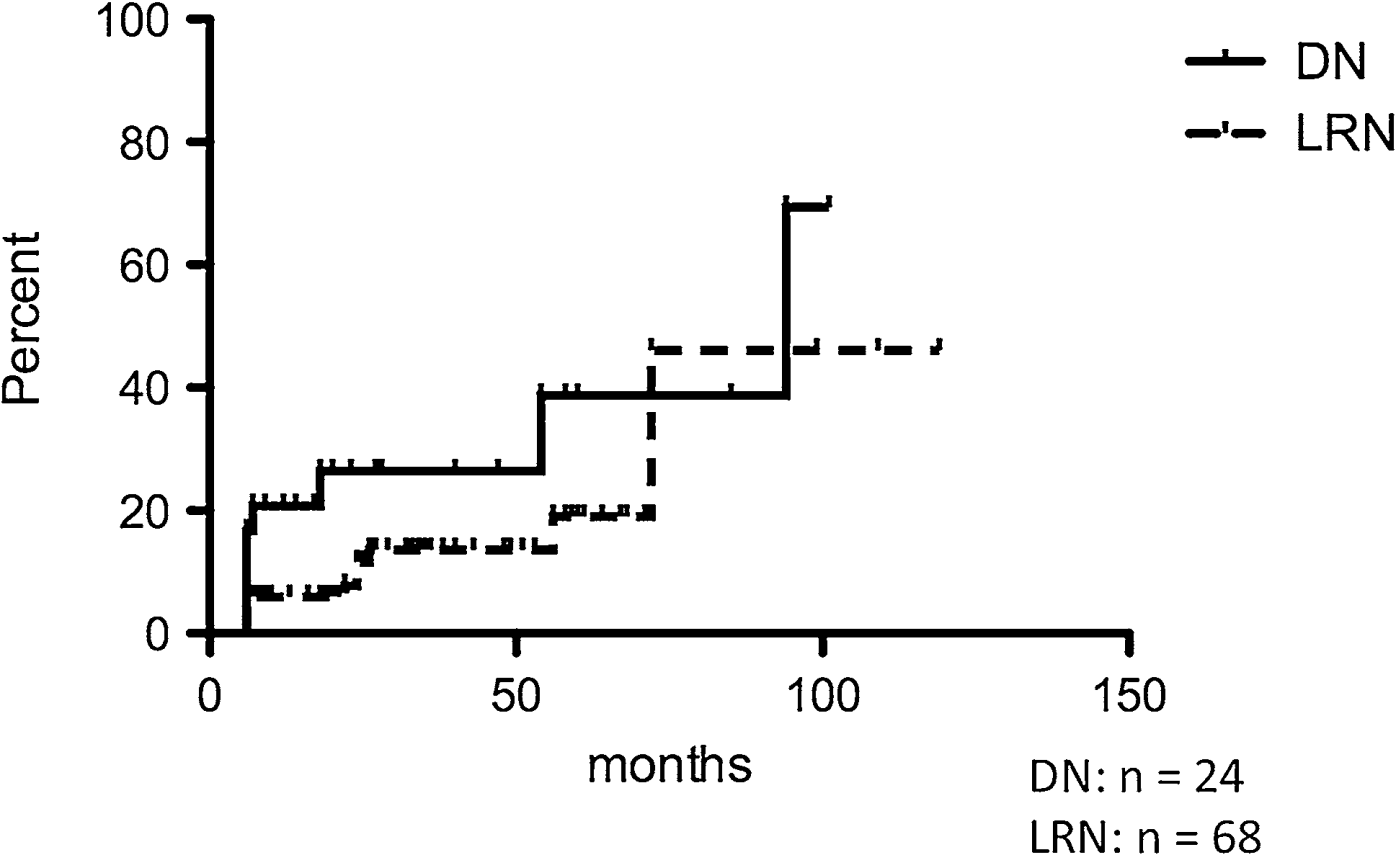
Rate of nodule enlargement. Nodule enlargement was more frequently found in dysplastic nodules than in large regenerative nodules, although the difference was not significant [*p* = 0.0743, Log-rank (Mantel–Cox) test]. *DN* dysplastic nodule, *LRN* large regenerative nodule

Figure 1 shows the rates of nodule size enlargement in both LRNs and DNs. Using the Kaplan–Meier method, the rates of enlargement at 50 months and 100 months were 13.7 and 46 % in LRNs and 26.5 and 69.4 % in DNs, respectively. Nodule enlargement was more frequently found in DNs than in LRNs, although the difference was not significant [*p* = 0.0743, Log-rank (Mantel–Cox) Test].

#### Rate of progression to malignancy

**Fig. 2:**
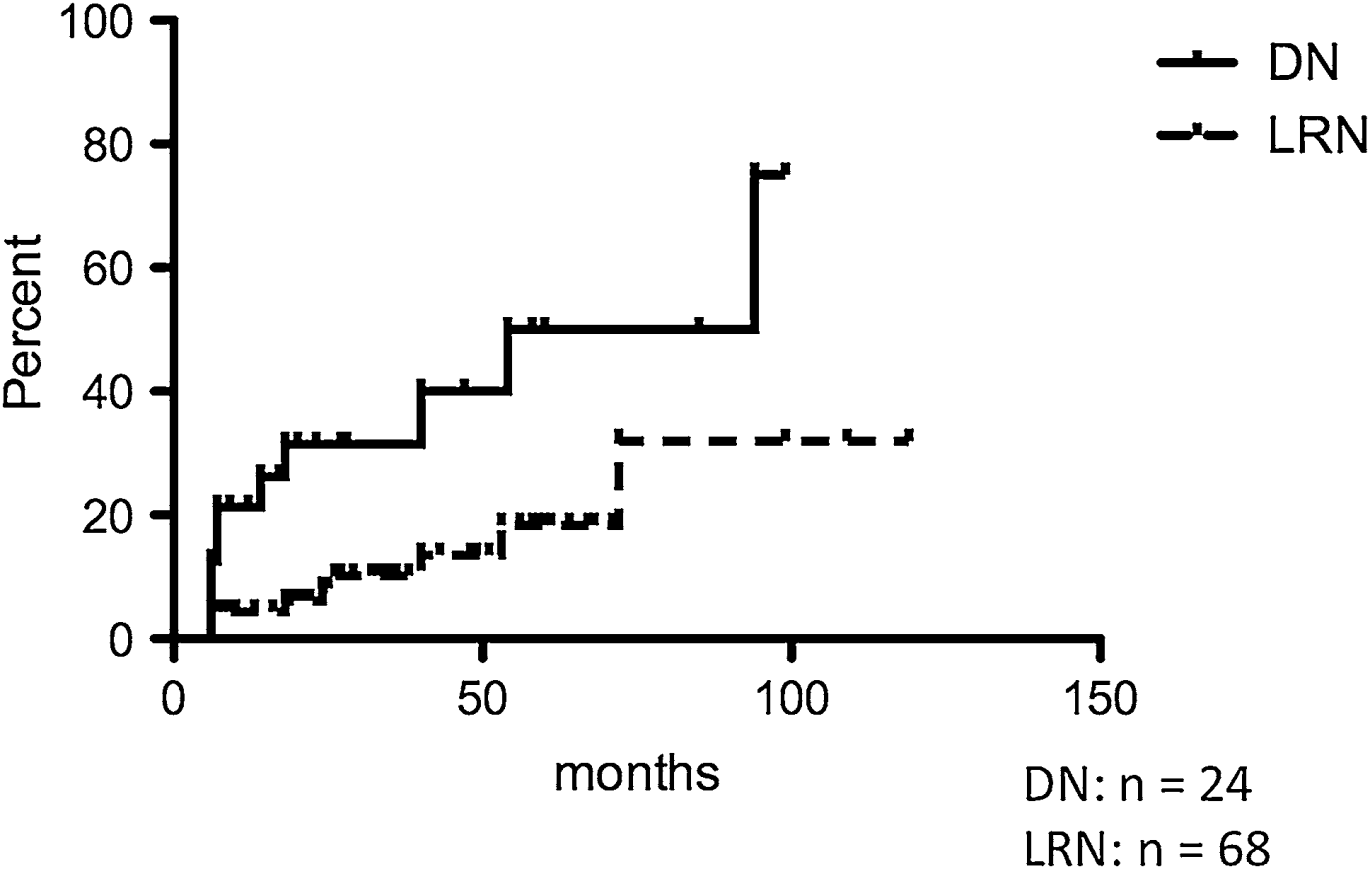
Rate of progression to malignancy. Malignant transformation was more frequently found in dysplastic nodules than in large regenerative nodules. The difference was statistically significant [*p* = 0.0040, Log-rank (Mantel–Cox) Test]. *DN* dysplastic nodule, *LRN* large regenerative nodule

The rates of progression to malignancy in LRNs and DNs are shown in Fig. 2. The rate of LRNs to HCC was 13.6 % at 50 months and 32 % at 100 months, while that of DNs was 40 % at 50 months and 75 % at 100 months, using the Kaplan–Meier method. Progression to malignancy was more frequently found in DNs than in LRNs, with the difference being statistically significant [*p* = 0.0040, Log-rank (Mantel–Cox) Test].

**Fig. 3:**
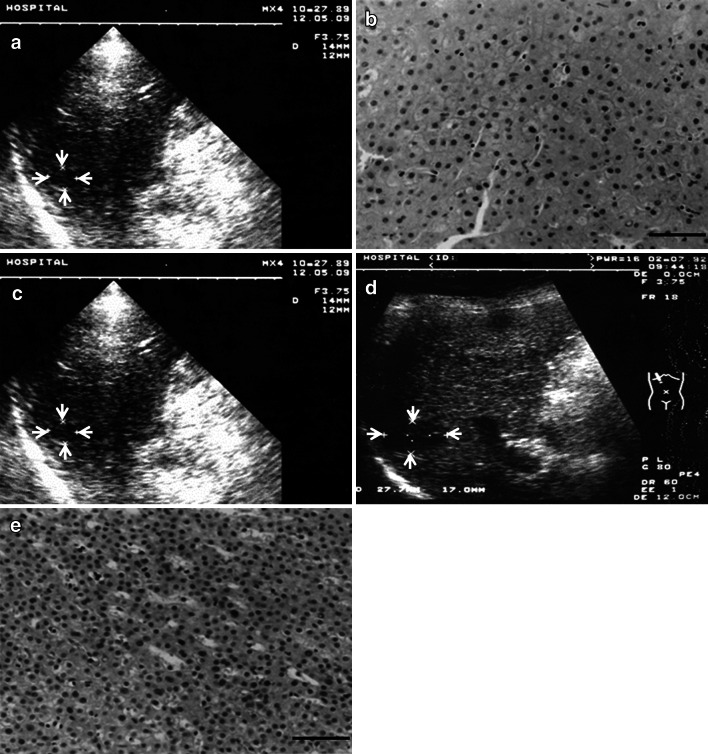
An LRN case showing malignant transformation. **a** A hypoechoic nodule 13 mm in diameter was detected by US (*arrows*). **b** Biopsy specimen showed minimal atypia. Diagnosis was large regenerative nodule (*bar* 50 μm). **c** Size and US findings showed no change for 26 months (*arrows*). **d** At 32 months, the nodule had enlarged to 23 mm (*arrows*). **e** Histological specimen from rebiopsy showed definite increased cellularity, and diagnosis was well differentiated HCC (*bar* 50 μm)

Figure 3 shows a representative case of proven malignant transformation of LRN. The patient was a 60-year-old male, HCV antibody-positive, with liver cirrhosis. A hypoechoic nodule 13 mm in diameter was detected by US (Fig. 3a). Biopsy diagnosis was a large regenerative nodule (Fig. 3b). The nodule showed no change in size and character for 26 months (Fig. 3c), but at 32 months, the nodule had grown to 23 mm (Fig. 3d). Rebiopsy revealed its progression to well differentiated HCC (Fig. 3e). This LRN kept a benign nature for 26 months and then progressed to HCC within another 6-month period.

Comparisons of initial profiles including age, serum blood test and tumor markers in cases with and without malignant transformation of nodules are shown in Table 3. Among these items, only one item, i.e. DN/LRN, was proven to be related to malignant transformation (*p* = 0.0066, Fisher’s exact test). Other items such as, nodule size, imaging findings, age, liver function tests, etiology of liver cirrhosis, tumor markers and association of DM were not related to malignant transformation.

**Table 3:** Comparisons of initial profiles in cases with and without malignant transformation of nodules

Parameter	Malignant	Non-malignant	*p* value
Nodule, *n*	19	73	–
Nodule size (mm)	12 (8–16)	11 (8–20)	0.8644^a^
US, hypo/hyper	13/6	54/9	0.7727^b^
DN/LRN	10/9	14/59	0.0066^b^
Age	63 (24–73)	67 (37–79)	0.4719^a^
AST (IU/ml)	60 (30–197)	73 (15–400)	0.1835^a^
ALT (IU/ml)	58 (11–466)	71 (11–466)	0.1835^a^
Platelet (×10^4^/μl)	11.7 (7.2–20.9)	11.2 (3.7–16.7)	0.6477^a^
Child classification/NBNC	12/4/3	54/11/8	0.9680^c^
AFP (ng/ml)	12.5 (3.1–108.9)	7.9 (1.8–657)	0.5505^a^
PIVKA-II (mAU/ml)	18 (<10–24)	15 (<10–31)	0.9382^a^
HCV/HBV/NBNC	12/4/3	54/11/8	0.6469^c^
DM (+)/(−)	3/16	8/65	0.6912^b^
Pre-HCC (+)/(−)	1/18	10/64	0.2710^b^

Data were shown as median and range

^a^Mann–Whitney test

^b^Fisher’s exact test

^c^Chi-square test

#### Disappearance on ultrasonography

**Fig. 4:**
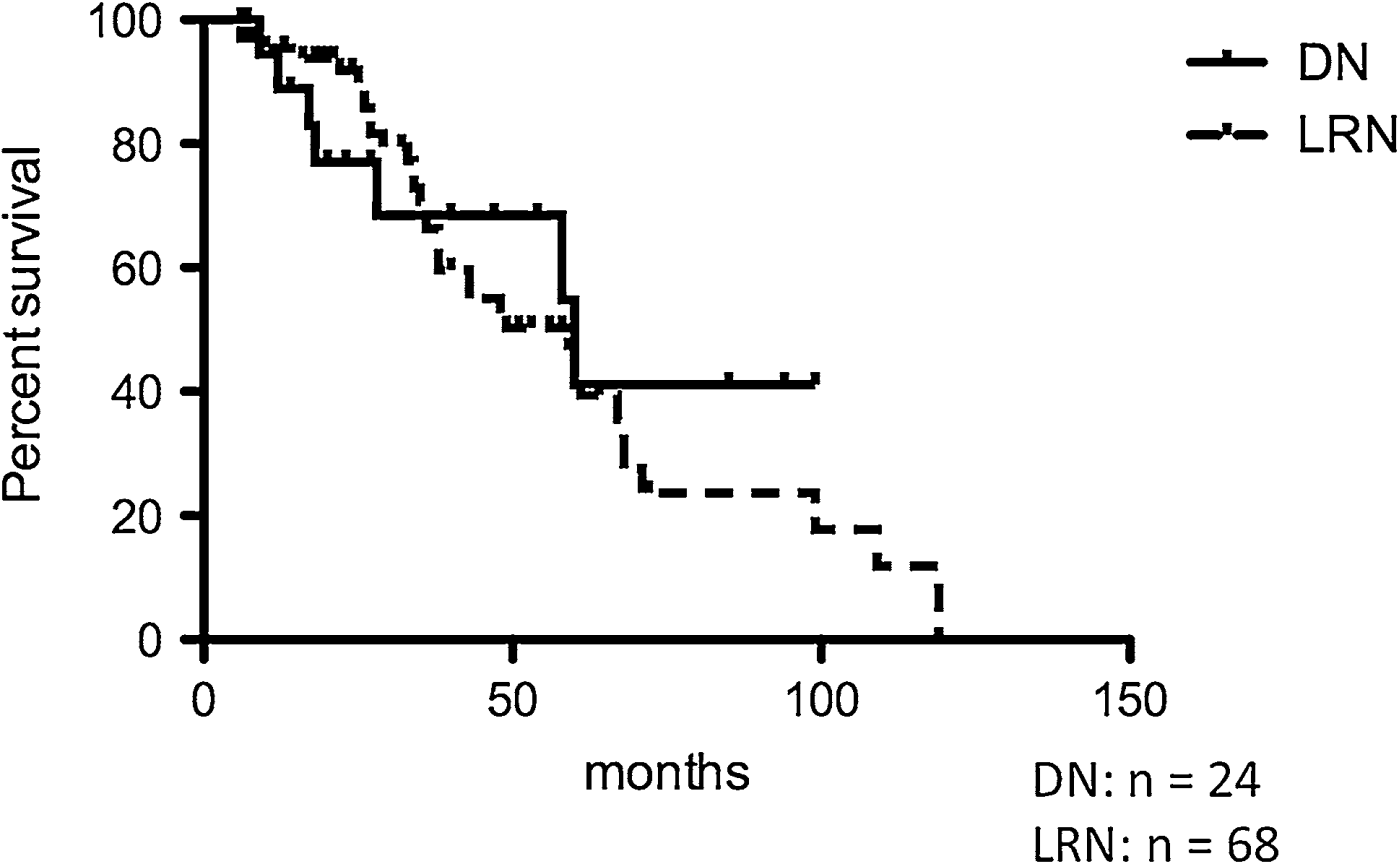
Rate of detection on ultrasonography. Rate of detection on ultrasonography was not significantly different in dysplastic nodules and large regenerative nodules [*p* = 0.6037, Log-rank (Mantel–Cox) test]. *DN* dysplastic nodule, *LRN* large regenerative nodule

Figure 4 shows the rate of detection of LRNs and DNs by US. During the follow-up period, some LRNs and DNs became undetectable by US, and they were also not detectable by other imagings (CT, MRI). The detection rates of nodules at 50 and 100 months were 50.3 and 17.8 % in LRNs and 68.5 and 41.1 % in DNs, according to the Kaplan–Meier method. There was no significant difference between LRNs and DNs. Various clinical data including DM, liver function tests and tumor markers shown in the Tables 1 and 3 were not proven to be related to the disappearance of the nodules.

#### Occurrence of new HCC lesions remote from nodules

Table 4 shows the frequency of occurrence of new HCC lesions remote from the followed-up nodules. The rate was 15 % (9 of 60 cases) in LRN cases and 22.7 % (5 of 22 cases) in DN cases. The difference was not statistically significant (*p* = 0.4100, Chi-square test).

**Table 4:** Occurrence cases of new HCC lesions remote from the nodules

Parameter	LRN	DN	*p* value
Occurrence of new HCC (+)	9	5	0.4100
Occurrence of new HCC (−)	51	17	

The occurrence of new HCC lesions was also reported in our previous study, and was thus confirmed in the present study.

## Discussion

A better knowledge of the natural history of small hepatic nodules including LRN, DN and HCC is important for the management of cirrhotic patients. We formerly investigated the natural history of HCC [12]. Although DN has been recognized as a pre-malignant lesion of HCC, the pre-malignant potentiality of LRN had not been clearly described in a number of important publications [10, 11, 13–15]. In fact, our previous study also did not confirm its pre-malingnant nature. Seventeen LRNs did not progress to HCC, and four of them became undetectable by various imagings. Four new HCC lesions developed remotely from the target LRN lesions [1].

However, we were not totally convinced of the result, as the number of LRNs and the lengths of follow-up periods were thought to be insufficient. Therefore, we continued the follow-up study after the previous report was published, and a greater number of LRNs were followed up for longer periods than before. In addition, DNs were followed up for comparison. We also wanted to confirm other results of the previous study, such as the disappearance of lesions in images and the occurrence of new HCCs remote from the target nodules.

The results showed that we could prove the pre-malignant nature of LRNs (Figs. 2 and 3). Although the rate was lower than that of DN, nine of 68 LRNs did show malignant transformation in the present study, quite a different result from our former study [1]. The reason for the difference must simply be attributable to the increases in the number of LRNs and follow-up periods. The difference in the rates of malignant transformation between LRN and DN is also important, highlighting the merit of classifying non-HCC lesions in cirrhotic liver into LRNs and DNs. We should recognize DNs as high risk lesions and LRNs as low risk lesions.

Except for the pre-malignant nature of LRNs, other phenomena described in our previous report were confirmed in the present study. Disappearance of nodules in images was found in both LRNs and DNs, although the reason for this can still not be conclusively described. However, chronic inflammation and structural change could be involved, with the former perhaps causing new fibrogenesis. The newly-formed fibrous septa would then have separated the nodules into smaller nodules. In any event, the mechanism of disappearance still needs to be clarified.

The occurrence of new HCC lesions remote from target nodules was also confirmed in the present study. As discussed previously [1], the new HCC lesion is considered to have developed from de novo-type carcinogenesis, bypassing a rather persistent stage of pre-cancerous nodules. Understanding this phenomenon is a critical issue in daily practical clinical activities [15]. In follow-up examinations, the whole liver should also be carefully examined, as well as these target nodules.

Finally, we hope the findings and considerations of this study will prove useful for practical clinical activities in the management of cirrhotic patients.

## Abbreviations

LRN: Large regenerative nodule
DN: Dysplastic nodules
HCC: Hepatocellular carcinoma
US: Ultrasound
CT: Computed tomography
MRI: Magnetic resonance image
HCV: Hepatitis C virus
HBV: Hepatitis B virus
